# Weather conditions associated with autumn migration by mule deer in Wyoming

**DOI:** 10.7717/peerj.1045

**Published:** 2015-06-23

**Authors:** Chadwick D. Rittenhouse, Tony W. Mong, Thomas Hart

**Affiliations:** 1 Department of Natural Resources and the Environment, Wildlife and Fisheries Conservation Center, University of Connecticut, Storrs, CT, USA; 2 Wyoming Game and Fish Department, Savery, WY, USA; 3 Environmental Services Section, Wyoming Department of Transportation, Cheyenne, WY, USA

**Keywords:** Camera trap, Wildlife underpass, Sagebrush steppe, Migration, *Odocoileus hemionus*, Mule deer

## Abstract

Maintaining ecological integrity necessitates a proactive approach of identifying and acquiring lands to conserve unfragmented landscapes, as well as evaluating existing mitigation strategies to increase connectivity in fragmented landscapes. The increased use of highway underpasses and overpasses to restore connectivity for wildlife species offers clear conservation benefits, yet also presents a unique opportunity to understand how weather conditions may impact movement of wildlife species. We used remote camera observations (19,480) from an existing wildlife highway underpass in Wyoming and daily meteorological observations to quantify weather conditions associated with autumn migration of mule deer in 2009 and 2010. We identified minimal daily temperature and snow depth as proximate cues associated with mule deer migration to winter range. These weather cues were consistent across does and bucks, but differed slightly by year. Additionally, extreme early season snow depth or cold temperature events appear to be associated with onset of migration. This information will assist wildlife managers and transportation officials as they plan future projects to maintain and enhance migration routes for mule deer.

## Introduction

Maintaining ecological integrity necessitates a proactive approach of identifying threats to species, communities, and the ecological processes that sustain them. For mule deer (*Odocoileus hemionus*), a culturally and economically important species (Copeland et al., 2014), habitat fragmentation can present barriers to migration, alter migration routes, or increase mortality through deer-vehicle collisions (Sawyer, LeBeau & Hart, 2012).

Highway underpasses have restored connectivity for mule deer in fragmented landscapes (Reed, Woodward & Pojar, 1975; Ng et al., 2004; Clevenger & Waltho, 2005; Braden et al., 2008; Gagnon et al., 2011). The unique structure of highway underpasses and associated fencing, when coupled with remote cameras and weather observations, presents an opportunity to gain substantial information on population age and sex structure (Ikeda et al., 2013), abundance (Rowcliffe et al., 2008), and proximate cues associated with migratory movement. Yet, the relationships between migratory movements and weather conditions are understudied, despite the importance of this for informing policy and adaptive management decisions regarding connectivity in the context of a changing climate.

Our objective was to identify weather conditions associated with autumn migration by mule deer in Wyoming. Our central hypothesis is that entering the winter range too early comes at the expense of reproductive output and survival of young mule deer. Our basis for this hypothesis arises from known nutritional limitations and energetic costs of winter to mule deer (Bartmann, White & Carpenter, 1992; Poole & Mowat, 2005; Bishop et al., 2009); premature entry to the winter grounds presumably provides no advantage to adults or offspring. Based on this hypothesis, we predict that mule deer movements through the underpass will be associated with the timing of snowfall events and snow depth that cover forage on summer grounds. Identification of the specific cue(s) used by mule deer during migration may allow managers to anticipate changes in migration based on weather conditions.

## Methods

### Study site and camera set-up

**Figure 1:**
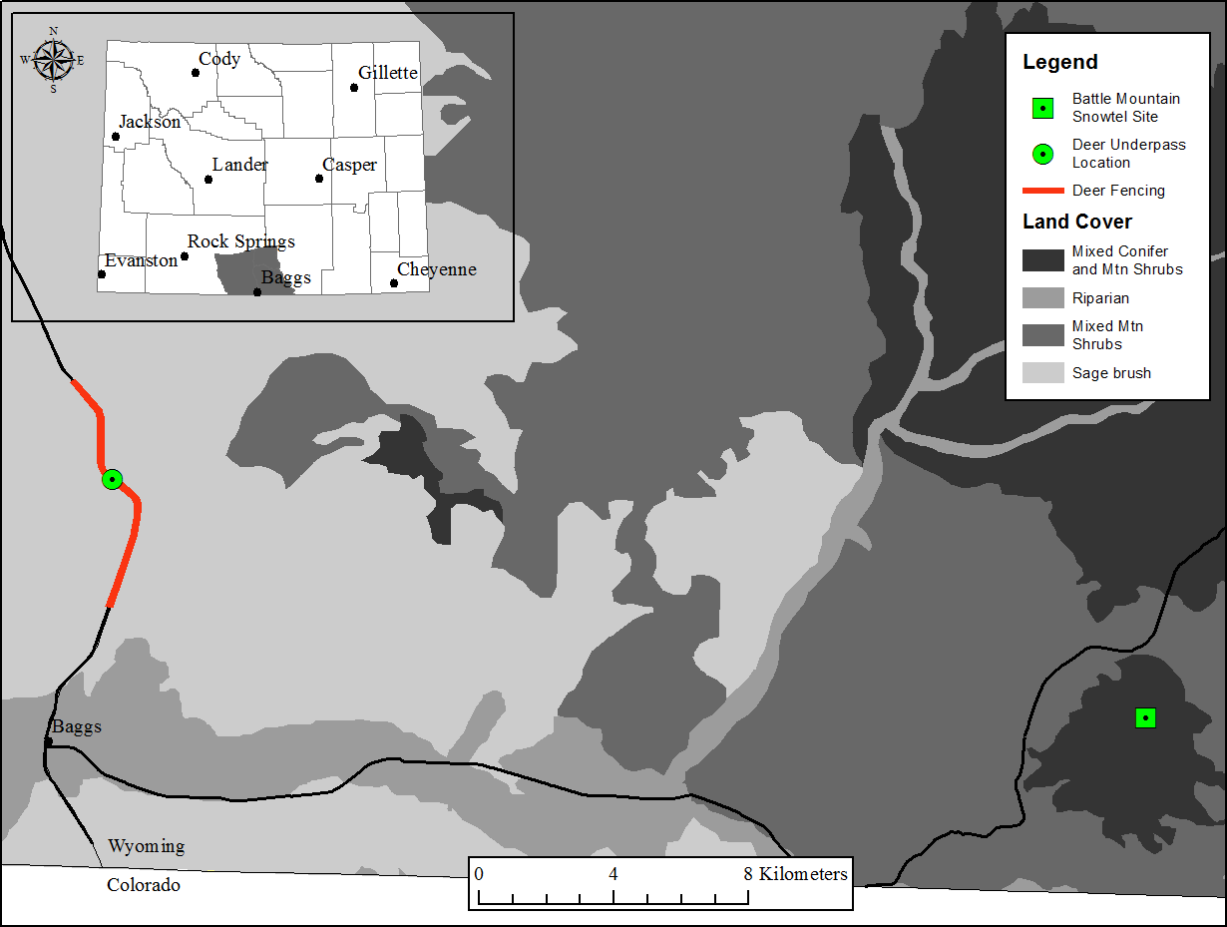
Study area near Baggs, Wyoming. Location of the highway underpass and fencing, and the meteorological station, near Baggs, Wyoming. During autumn, mule deer migrate from the higher elevation summer range north and east of the underpass to lower elevation winter range to the west of the highway that contains the underpass.

We collected animal migration data using a trail camera set up to monitor a highway underpass on Hwy. 789 approximately 8 km north of Baggs, Wyoming (Fig. 1). The underpass was installed in 2009 and equipped with a RECONYX Hyperfire (TM) camera (Reconyx, Holmen, Wisconsin, USA) mounted approximately 1.5 m high centered within the underpass. The camera was pointed towards the direction animals were migrating from, so during autumn the camera was pointed east. Camera settings included a distance from camera to subjects of 18.2 m with a 1/5 s trigger speed; three photos recorded when motion was detected, and photo resolution of 1080P High Definition or 3.1 Mega-pixels. Cameras remained active throughout the year; however, during autumn migrations by mule deer the images were downloaded more frequently. Autumn migration dates were November 1 to December 31 of each year. The Wyoming Game and Fish Department approved this research (Permit #791).

The study site was defined by the Baggs Mule Deer Herd Unit, which encompasses 1,092 km^2^ south of I-80 in southern Carbon County (Fig. 1). This area supports a variety of vegetation types, but is generally characterized by rolling topography, prominent ridges, and dry canyons dominated by sagebrush (*Artemisia sp.)*, black greasewood (*Sacrobatus vermiculatus*), Utah juniper (*Juniperus osteosperma*), and other mixed-shrub (*Purshia tridentata, Prunus virginiana, Amelanchier alnifolia, Chrysothamnus sp., Cercocarpus sp.*). Elevations range from 1,920 to 2,530 m.

### Population age and sex structure

We used images taken by the camera to count and assign age class (fawn, yearling, adult) and sex to all deer passing through the underpass during autumn migrations 2009 and 2010. Does and fawns migrated together, as did adult and yearling bucks. Therefore, we tallied does separate from fawn, yearling buck, and adult classes. While most deer moved through the underpass one way, in some instances multiple images were obtained of the same individuals due to the three-image sequence provided by the camera. When this occurred, we used group size, group composition, and size of individuals to count unique individuals only.

Common indices used by wildlife managers to monitor population sex and age structure include: ratio of adult doe per fawn, ratio of adult doe per yearling buck, and ratio of adult doe per adult buck. We quantified those ratios for fall migration of each year and provide SE and 95% CI.

### Association of migratory movements with weather conditions

We obtained meteorological records from the Battle Creek weather station (31 km from underpass, Natural Resources Conservation Service Snotel Site number 317, 41°; 3 min N, 107°; 16 min W). The meteorological records consisted of daily records of maximum air temperature (°C), minimum air temperature (°C), average air temperature (°C), precipitation accumulation (in), snow water equivalent (in), and snow depth (in). From those weather observations, we calculated one additional metric: snow event. Snow event had a value of 1 if snow fell on that day. If no snow fell on that day we assigned a value of 0 to snow event. Preliminary analyses indicated high correlations (*R* > 0.50) among all weather variables, so we proceeded with models containing each weather variable separately. Preliminary analyses also indicated that weather observations from the previous day were better predictors of daily migration counts than same day observations because they accounted for the lag between a weather event at higher elevation and deer arrival at the underpass. Therefore, all weather conditions except snow depth reflected the previous day’s weather.

To identify weather conditions associated with autumn migration by mule deer, we modeled counts of does and bucks (yearling and adult combined) as a function of weather variables. The counts were specified as the response variable in separate models of minimum air temperature, maximum air temperature, precipitation amount, snowfall event, and snow depth as independent variables. For each model, we included each independent weather variable and an interaction term with day of the migration season, with day 1 corresponding to 17 October 2009 and 8 October 2010. We also examined a model that consisted of all independent variables and their interactions with day of migration season.

We used negative binomial regression models to handle overdispersed count data with 0 observations. We used Akaike’s information criterion (AIC) to rank models and Akaike weights (*w_i_*) to determine which model associating migration with weather conditions had the strongest support (Burnham & Anderson, 2002). We fitted all models with the glm.nb function in the R language and environment for statistical analyses (version 2.15.2) (R Core Development Team, 2012).

## Results

Analysis of 19,480 images acquired during fall migration 2009 and fall migration 2010 documented 6,628 counts of mule deer using the underpass (Table 1). The population age and sex structure was consistent across the two years with a mean of 61 fawns, 8 yearling bucks, and 14 adult bucks per 100 does (Table 1).

**Table 1:** Counts of mule deer by age and sex classes. Age and sex counts and ratios of mule deer in the Baggs District of Wyoming observed from a camera trap fixed to a highway underpass during autumn migration of 2009 and 2010.

Year	Sex-age class	*N*	Ratio of does per sex-age class	SE	−95% CI	+95% CI
2009	Does	1,205				
	Fawns	754	1.60	0.07	1.45	1.74
	Yearling bucks	81	14.88	1.71	11.53	18.22
	Adult bucks	170	7.09	0.58	5.95	8.23
2010	Does	2,401				
	Fawns	1,441	1.67	0.06	1.56	1.78
	Yearling bucks	228	10.53	0.73	9.10	11.96
	Adult bucks	315	7.62	0.46	6.73	8.52

Model-selection results indicated minimum air temperature and snow depth were the best proximate cues associated with autumn migration. In 2009, minimum air temperature of the previous day was the most supported model for does and bucks, with maximum air temperature of the previous day competing with the most supported model for bucks only (Table 2). In 2010, snow depth was the most supported model for does and bucks with no competing models for either sex. Higher counts of deer occurred on days with lower minimum temperatures in 2009 and on days with greater snow depth in 2010 (Table 3). Bucks and does responded similarly to weather conditions within and across years despite migrating in separate groups.

**Table 2:** AIC ranks for models associating weather and mule deer migration. Ranked empirical support for models examining how weather conditions influence autumn migration by mule deer in the Baggs District of Wyoming. All models included day as a variable, but the variable name was omitted for brevity. Data collected from a camera trap fixed to a highway underpass during autumn migration of 2009 and 2010.

Sex	Year	Model	AIC	Δ AIC	*w_i_* ^a^	*r* ^2^ ^b^
Does	2009	Min air temp, previous day	517.73	0.00	0.83	0.42
		Max air temp, previous day	521.10	3.36	0.15	0.39
		Full model	525.76	8.03	0.02	0.39
		Snow depth	540.58	22.85	0.00	0.19
		Snowfall event, previous day	548.84	31.11	0.00	0.08
		Precip. amount, previous day	550.21	32.47	0.00	0.06
	2010	Snow depth	616.34	0.00	0.95	0.58
		Min air temp, previous day	622.12	5.79	0.05	0.55
		Max air temp, previous day	629.18	12.85	0.00	0.50
		Snowfall event, previous day	647.14	30.81	0.00	0.37
		Precip. amount, previous day	650.81	34.48	0.00	0.34
		Full model	655.28	38.94	0.00	0.33
Bucks	2009	Min air temp, previous day	329.55	0.00	0.43	0.26
		Max air temp, previous day	329.87	0.32	0.37	0.26
		Snow depth	332.39	2.84	0.10	0.23
		Full model	332.49	2.94	0.10	0.27
		Snowfall event, previous day	344.95	15.39	0.00	0.07
		Precip. amount, previous day	348.70	19.15	0.00	0.02
	2010	Snow depth	405.98	0.00	1.00	0.58
		Min air temp, previous day	426.15	20.17	0.00	0.46
		Max air temp, previous day	434.34	28.36	0.00	0.39
		Full model	446.80	40.82	0.00	0.32
		Snowfall event, previous day	448.51	42.53	0.00	0.27
		Precip. amount, previous day	452.30	46.31	0.00	0.23

**Notes.**

a Weights of evidence.

b Fitted model versus null model (Magee, 1990).

**Table 3:** Influence of weather on autumn migration by mule deer. Parameter estimates, standard errors, *z* scores, and *p*-values for the most-supported models examining how weather conditions influence autumn migration by mule deer in the Baggs District of Wyoming. Data collected from a camera trap fixed to a highway underpass during autumn migration of 2009 and 2010.

Sex	Year	Parameter	Estimate	Std. error	*z* value	*P*-value
Does	2009	Intercept	2.217	0.245	9.065	<0.001
		Min air temp, previous day	−0.206	0.038	−5.364	<0.001
		Day	0.011	0.009	1.193	0.233
		Min air temp, previous day:Day	0.004	0.001	3.719	<0.001
	2010	Intercept	1.229	0.253	4.854	<0.001
		Snow depth	0.371	0.057	6.562	<0.001
		Day	0.052	0.010	5.426	<0.001
		Snow depth:Day	−0.007	0.001	−7.328	<0.001
Bucks	2009	Intercept	1.300	0.272	4.773	<0.001
		Min air temp, previous day	−0.127	0.042	−3.044	0.002
		Day	−0.011	0.010	−1.091	0.275
		Min air temp, previous day:Day	0.002	0.001	1.409	0.159
	2010	Intercept	0.571	0.238	2.401	0.02
		Snow depth	0.402	0.047	8.487	<0.001
		Day	0.026	0.009	2.945	0.003
		Snow depth:Day	−0.007	0.001	−7.910	<0.001

**Figure 2:**
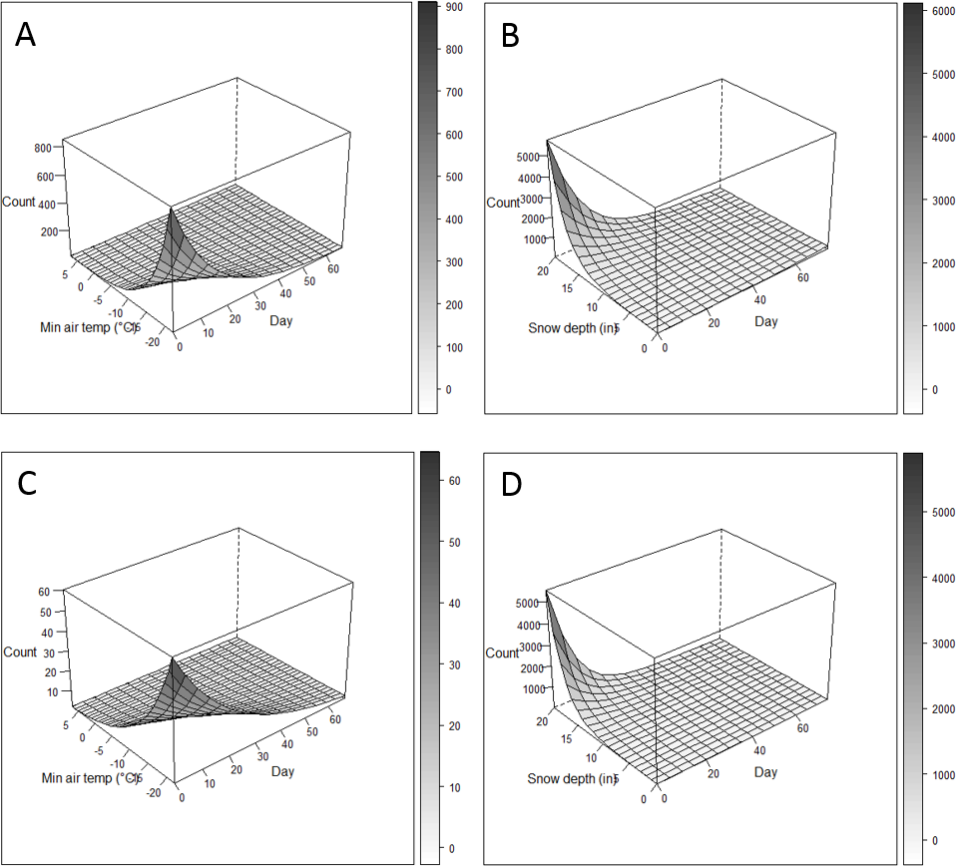
Predicted use of a highway underpass based on weather conditions. Predicted use of highway underpass during autumn migration based on most supported models of doe (A, B) and buck (C, D), over the range of weather conditions observed in 2009 (A, C) and 2010 (B, D) near Baggs, Wyoming.

We used coefficients from the top fitted models to predict use of the highway underpass under the range of weather conditions observed in 2009 and 2010 (Fig. 2). Early, extreme minimum air temperatures and snow depths had the highest predicted counts of does and bucks. Based on these model predictions, thresholds for onset of migration were minimum air temperature of 0 to −5 °C or snow depth exceeding 25.4 cm.

## Discussion

Wildlife underpasses and overpasses are used throughout western North America to restore connectivity for migratory and large-ranging species. We demonstrated that monitoring wildlife underpasses provides important demographic and movement information, which when coupled with weather observations can be used to understand connectivity and migratory dynamics associated with weather conditions. The association between weather conditions and counts of deer using the underpass was consistent with our expectation. Specifically, we identified minimal daily temperature and snow depth as proximate cues used by mule deer during migration to winter range. These weather cues were consistent across does and bucks, but differed slightly by year. Additionally, extreme early season snow depth exceeding 25.4 cm or minimum air temperature of 0 to −5 °C may be associated with onset of migration.

Our results using a single camera trap were consistent with radio-telemetry studies of mule deer migration. Previous studies identified decreasing daily temperature, increasing snow depth, and senescing vegetation as factors associated with autumn migration from high-elevation summer ranges to low-elevation winter ranges (Garrott et al., 1987; Nicholson, Bowyer & Kie, 1997; Monteith et al., 2011). However, the specific relationships varied by vegetation type, elevation, and climate. In the sagebrush-steppe ecosystem of the Sierra Nevada Mountains in California, the onset of migration coincided with average daily temperature <5 °C and snow depth >0 cm (Monteith et al., 2011). In a pinyon pine-Utah juniper shrubland complex in northwestern Colorado, mule deer migration occurred with average daily temperature >0 °C and no snow depth (Garrott et al., 1987). In the San Bernardino Mountains of southern California, mule deer exhibited partial migration in response to snow cover (Nicholson, Bowyer & Kie, 1997).

Installation of under and overpasses are effective in reducing deer-vehicle collisions (Clevenger, Chruszez & Gunson, 2001). However, under and overpasses may be cost prohibitive and thus alternative methods of migration route protection may need to be developed. In this situation, variability in the number of mule deer entering the winter range poses substantial challenges to managing mule deer-human interactions. One alternative is to use weather information to better time the use of temporary signage on roads crossed by migration routes. Temporary signage could indicate speed reduction or simply increase awareness that migration movements are more likely on days with suitable weather conditions.

In addition to providing information that could be used to more effectively manage migration route-road crossings, acquiring images of deer using underpasses and overpasses may also increase the efficiency of monitoring efforts by state agencies. Long-term population monitoring for setting harvest regulations typically uses aerial surveys of summer grounds conducted just after the hunting season and prior to migration to wintering grounds. A concern with post-hunt aerial surveys is the potential to miss animals or have a biased sample if some classes of animals migrate prior to the survey. In these situations, underpass and overpass data may complement post-hunt aerial surveys by providing additional sampling periods during spring and fall migration. Having multiple sampling periods would enable within-year estimates of survival and recruitment as opposed to a single, annual estimate.

## Conclusions

Underpass and overpass data coupled with meteorological observations can be used to understand the relationship between weather and migration. With short-term data, we identified minimum daily temperature and snow depth as proximate cues associated with mule deer migration to winter range in Wyoming. Long-term data will provide information on migration dynamics, including whether the onset, duration, or magnitude of migration co-changes with weather. Long-term data may also reveal the role of weather in partial migration, cessation of migration or changes in route location over time.

## Supplemental Information

10.7717/peerj.1045/supp-1 Supplemental Information 1 Raw data of mule deer counts and weather conditions in 2009

10.7717/peerj.1045/supp-2 Supplemental Information 2 Raw data of mule deer counts and weather conditions in 2010

## Additional Information and Declarations

### Field Study Permissions

The following information was supplied relating to field study approvals (i.e., approving body and any reference numbers): The Wyoming Game and Fish Department approved this research (Permit #791).
